# Efficacy of *DigiKnowItNews: Teen*, a multimedia educational website for adolescents about pediatric clinical trials: study protocol for a randomized controlled trial

**DOI:** 10.1186/s13063-023-07464-0

**Published:** 2023-06-30

**Authors:** Alison E. Parker, Tracy M. Scull, Kathryn L. Kennedy

**Affiliations:** 1 innovation Research & Training, Durham, NC USA; 2 University of North Carolina-Chapel Hill, Chapel Hill, NC USA

**Keywords:** Adolescents, Pediatric clinical trials, Knowledge, Attitudes, Self-efficacy, Shared decision-making, Education

## Abstract

**Background:**

Pediatric research is crucial for the development of new scientific advancements, treatments, and therapies for adolescents. Yet relatively few pediatric clinical trials are conducted due to barriers to successful recruitment and retention, including knowledge and attitudes about clinical trials. Adolescents tend to experience greater autonomy to make decisions and have expressed interest in being part of the decision to participate in clinical trials. Increasing knowledge, positive attitudes, and self-efficacy related to clinical trials could positively impact the decision to participate in a pediatric clinical trial. However, there are currently few interactive, developmentally appropriate, web-based resources available to educate adolescents about clinical trials. *DigiKnowItNews: Teen* was created as a multimedia educational website to address the relatively low levels of enrollment in pediatric clinical trials and need for information to empower adolescents to make decisions about participating in clinical trials.

**Methods:**

This is a parallel group randomized controlled superiority trial to test the effectiveness of *DigiKnowItNews: Teen*, for improving factors related to clinical trial participation among adolescent and parents. Eligible parent-adolescent (ages 12 to 17 years) pairs will be randomly assigned to one of two conditions: intervention or wait-list control. All participants will complete pre- and post-test questionnaires and participants assigned to the intervention will receive access to review the *DigiKnowItNews: Teen* content for 1 week. Wait-list control participants will have the option to review *DigiKnowItNews: Teen* after study completion. The primary outcomes are knowledge about clinical research, attitudes, and beliefs toward pediatric clinical trials, self-efficacy for making decisions related to clinical trial participation, willingness to participate in a future clinical trial, procedural fears, and parent-adolescent communication quality. Overall feedback and satisfaction related to *DigiKnowItNews: Teen* will also be collected.

**Discussion:**

The trial will evaluate the effectiveness of *DigiKnowIt News: Teen*, an educational website about pediatric clinical trials for adolescents. If found effective in promoting factors related to future pediatric clinical trial participation, *DigiKnowIt News: Teen* could be used by adolescents, along with their parents, as they make the decision to participate in a clinical trial. Clinical trial researchers can also use *DigiKnowIt News: Teen* to aid their participant recruitment efforts.

**Trial registration:**

ClinicalTrials.gov NCT05714943. Registered on 02/03/2023.

## Administrative information

Note: the numbers in curly brackets in this protocol refer to SPIRIT checklist item numbers. The order of the items has been modified to group similar items (see http://www.equator-network.org/reporting-guidelines/spirit-2013-statement-defining-standard-protocol-items-for-clinical-trials/).

**Table:** 

Title {1}	Efficacy of *DigiKnowItNews: Teen*, a multimedia educational website for adolescents about pediatric clinical trials: Study protocol for a randomized controlled trial
Trial registration {2a and 2b}.	ClinicalTrials.gov, [NCT05714943]. Registered on [02/03/2023]
Protocol version {3}	This study was approved by iRT’s IRB Committee (IRB number: 22–002-01-EFF; May 24, 2022)
Funding {4}	Research reported in this publication was supported by the National Institute of Nursing Research of the National Institutes of Health under Award Number R44NR019565. The content is solely the responsibility of the authors and does not necessarily represent the official views of the National Institutes of Health.
Author details {5a}	^1^innovation Research & Training, Durham, NC; ^2^University of North Carolina-Chapel Hill, Chapel Hill, NC
Name and contact information for the trial sponsor {5b}	innovation Research & Training (iRT) 5316 Highgate Drive, Suite 125, Durham, North Carolina, USA 27713
Role of sponsor {5c}	iRT (as the sponsor) does not have a role in the study. Drs. Parker and Scull are both lead investigators for the study. Responsibilities will include supervising and managing data collection and data analyses, as well as writing and submitting the report for publication.

## Introduction

### Background and rationale {6a}

Scientific innovation and drug development for children and adolescents is predicated on pediatric clinical research [1, 2]. Yet relatively few pediatric clinical trials are performed, and some trials fail to meet recruitment goals [3, 4]. In 2022, only 6 out of 37 (16%) of the new drug and biologic applications approved by the FDA (Food and Drug Administration) included a pediatric indication for use [5]. As a result, health care professionals may prescribe medications whose results have been studied only in the adult population, and have not been tested for age-appropriate dosing, safety, and/or efficacy [1, 6]. This is especially problematic for children and adolescents since absorption and metabolism of drugs may differ by age and developmental status [7]. Thus, new clinical trials are needed to collect relevant data to effectively and safely manage pediatric diseases. The necessity for pediatric clinical trials has gained emerging interest and attention through recent legislation and policy change and the FDA’s efforts to highlight the critical role of pediatric clinical trials to ensure the safety and efficacy of drugs for children and adolescents [6, 7].

Despite the shift toward greater acceptance and acknowledgement of the importance of involving children and adolescents in drug development research, successful enrollment for pediatric clinical trials continues to be a long-standing challenge due to barriers to participant recruitment [8, 9]. Some barriers exist because of the smaller number of eligible and interested pediatric participants, concerns about study risks or procedures, randomization to different study arms, or travel and time burdens [4, 7, 8, 10, 11]. Additional barriers specific to adolescent participants include feeling overwhelmed with the quantity of information provided about a study, lacking awareness of and adequate information to properly understand the research, or experiencing mistrust or fear about the study [12–14]. However, some adolescents describe wanting more information and to be involved in the decision to be in a clinical trial [15, 17]. In addition, as adolescents grow older, shared decision-making with their parents may increase, and there may be more opportunities to address the perceived barriers during discussions about participation [15–19].

One way to help overcome these barriers is by providing information to potential clinical trial participants as they weigh the decision whether or not to be in a study [20]. This information may be presented in several different formats. For example, previous studies have demonstrated the positive effect of sharing information using multimedia and technology (e.g., computers, tablets) and its ability to improve knowledge and understanding related to clinical trial participation [21–24]. Educating adolescents and parents about pediatric clinical trials may impact their willingness to participate in clinical trials and potentially increase clinical trial recruitment and enrollment [13, 25].

*DigiKnowItNews: Teen (DKIN: Teen)* is an interactive, multimedia educational website for adolescents ages 12–17 years old that was created in response to the relatively low levels of enrollment in pediatric clinical trials and the need for information about clinical trials that is more relevant and engaging for adolescents. The goal of *DKIN: Teen* is to increase adolescent knowledge, attitudes, and efficacy toward clinical trial participation as well as parent-adolescent communication and shared decision-making related to clinical trial participation. A version of *DigiKnowIt News (DKIN)* was developed for children and evaluated in a randomized controlled trial; children who participated in *DKIN*, compared to those that did not, experienced an increase in knowledge related to clinical trials and confidence in their ability to participate in a clinical trial [26].

An educational website specifically for adolescents is needed to provide content that is relevant and tailored for older adolescents who are more likely to be making decisions both independently and jointly with their parents related to clinical trial participation. *DKIN: Teen* is developmentally appropriate, relevant, and relatable for adolescents by presenting user-friendly information in various interactive multimedia formats. The creation of *DKIN: Teen* builds off of previous evidence that adolescents may have the ability to competently engage in decision-making starting around age 12 [27]. By providing adolescents with content tailored to their unique perspectives and needs, as well as focusing on shared decision-making with their parents, *DKIN: Teen* has the ability to facilitate communication between parents and adolescents deciding whether to participate in a clinical trial.

The present study is a randomized controlled trial to test the effectiveness of *DKIN: Teen* related to several factors impacting clinical trial participation. This study seeks to examine if *DKIN: Teen* will increase adolescents’ knowledge about clinical trials, adolescents’ and parents’ positive attitudes and beliefs toward clinical trials, adolescents’ self-efficacy about the decision to participate in a clinical trial, and adolescents’ and parents’ likelihood and willingness to participate in a clinical trial. In addition, *DKIN: Teen* may promote communication among parents and adolescents deciding about whether to enroll in a clinical trial. In providing information and ensuring potential participants are well-informed, *DKIN: Teen* has the ability to support and empower adolescents and their parents to make the decision about whether to participate in a clinical trial.

### Objectives {7}

The primary objective of the study is to evaluate the effectiveness of *DKIN: Teen* in a randomized controlled trial for impacting parent and adolescent knowledge, attitudes, self-efficacy, and fear related to pediatric clinical research, likelihood and willingness to participate in a clinical trial, and parent-adolescent communication.

We hypothesize that adolescents who use *DKIN: Teen*, compared to those who do not, will have increased knowledge about clinical trials, more positive attitudes about clinical trials, greater self-efficacy about making an informed decision to participate in clinical research, greater likelihood and willingness to participate in future clinical trials, better parent-adolescent communication quality, and fewer fears about research procedures. We hypothesize that parents, who use *DKIN: Teen*, compared to those that do not, will have more positive attitudes and beliefs about clinical trials, greater likelihood and willingness to participate in future clinical trials, and better parent-adolescent communication quality.

### Trial design {8}

The present study is a parallel group randomized controlled trial with a 1:1 allocation ratio and superiority framework to test the effectiveness of *DKIN: Teen* for improving factors related to clinical trial participation among parent and adolescent pairs. Participants will be randomly assigned to one of two conditions: (1) intervention or (2) wait-list control.

## Methods: participants, interventions and outcomes

### Study setting {9}

The study will be conducted online and data will be collected in the USA. Interested parents of adolescents will visit the study recruitment website to learn more about the study and respond to online eligibility screening questions. If eligible, parents and adolescents will complete online consent and permission and assent forms. All trial procedures will take place online; participants will access study questionnaires and *DKIN: Teen* on their own electronic device (e.g., computer, tablet) with an Internet connection. Communication with the study team will take place via email with an option to also receive communication via text message. Participants will receive links via email (and text if opting in) to complete the pre- and post-test questionnaires and access *DKIN: Teen*. Project staff members will monitor *DKIN: Teen* usage remotely through the Learning Management System (LMS).

### Eligibility criteria {10}

Inclusion criteria include (1) adult participants are a parent or legal guardian of adolescent participant, (2) adolescent participants are within the ages of 12–17 years, (3) parent-adolescent pairs have access to a computer or tablet with internet connection for the duration of the study, and (4) parent-adolescent pairs are fluent (able to read and write) in English. Exclusion criteria include if an adolescent participant has previously participated in a clinical trial after kindergarten. Eligible parent and adolescent pairs will be informed that in an attempt to have a diverse sample, they may not be invited to participate.

### Who will take informed consent? {26a}

Informed consent will be self-administered and completed online through the study recruitment website. Upon completing online eligibility questions, parents will be notified onscreen immediately if the parent-adolescent pair is eligible or not to participate in the study. If a parent-adolescent pair is eligible, they will see a screen that states that they can follow a link to complete the study forms. Parents will complete an online consent form for their own participation and permission form for their child’s participation and the adolescent will be asked to complete an online assent form. Parents and adolescents will be instructed to review the forms together so they can decide about whether they want to participate. The consent forms will include contact information for the leading investigators and IRB chair in case they have any questions related to the study. Parent-adolescent pairs who do not complete the study forms will not be eligible for study enrollment.

### Additional consent provisions for collection and use of participant data and biological specimens {26b}

This trial does not involve collecting biological specimens for storage. There are no ancillary studies that require additional consent provisions.

### Interventions

#### Explanation for the choice of comparators {6b}

The comparator is the wait-list control group who will not have access to the intervention *(DKIN: Teen)* between the pre-test and post-test questionnaires. After completing the post-test questionnaires, participants in the wait-list control group will be given the option to access *DKIN: Teen.* The comparator aligns with the main aim of the study, which is to determine the effectiveness of *DKIN: Teen* for improving factors related to pediatric clinical trial participation among adolescents.

### Intervention description {11a}

The intervention group will receive access to the intervention, *DKIN: Teen*, which is an interactive, multimedia educational website that provides high-quality information to educate adolescents and parents about pediatric clinical trials and provide resources for communication and shared decision-making about research. *DKIN: Teen* includes multiple ways to learn about clinical trials, such as interactive activities, animations, and videos, so that adolescents can select how they want to engage with the website based on their own abilities and interests. The website is organized into four main areas: Investigations, Spotlights, Comic Books, and Shared Decision-Making.

- The Investigations are interactive learning modules with educational information about clinical trials in general (e.g., rights, roles, safety) and specific procedures that might be used in clinical trials (e.g., needles, scans, organ function testing). Each of the modules contains six small sections with information presented in various formats: key terms and definitions, fun facts, animations, interactive activities, videos of peers or health professionals, and real-life scenario videos depicting clinical trial experiences.
- The Spotlights section includes video testimonials of adolescents sharing their own experiences with participating in pediatric clinical trials about various topics related to definitions (e.g., assent), communication, decision-making, advice, and procedures (e.g., MRIs).
- The Comic Books section includes fictitious clinical trial storylines in which adolescents select a character to follow throughout while they interact with the comic.
- The Shared Decision-Making section includes an interactive learning module for adolescents and their parents to learn about and practice steps for shared decision-making.

*DKIN: Teen* is optimized for use on computers and tablets, making it convenient and portable for adolescents. The website is also accessible by screen readers; video and animation content include closed captioning. *DKIN: Teen* will be self-administered online and completed by users at their own pace over the period of a week.

### Criteria for discontinuing or modifying allocated interventions {11b}

As this is a low-risk study, there are no specific criteria for discontinuing or modifying allocated interventions for participants. Participants may choose to stop participating in the intervention or study for any reason and will be informed on the permission, consent, and assent forms that they can choose to stop participating in the study at any time without consequence.

### Strategies to improve adherence to interventions {11c}

Participants will receive automatic email (and text if opted in) reminders from the study management system if they have not completed the pre- or post-test questionnaires or have not accessed *DKIN: Teen*. A study team member will also check in directly with the participating parent if there is no response following the automatic reminders.

### Relevant concomitant care permitted or prohibited during the trial {11d}

Participants will continue usual care and medications throughout the trial. There are no care or interventions prohibited during the trial.

### Provisions for post-trial care {30}

This is a low-risk study, and it is not anticipated that there will be a need for post-trial care. However, the contact information for the IRB chair and the leading investigators is listed on the study forms if needed by participants during or after the study.

### Outcomes {12}

The primary outcomes will be measured at baseline and week 1. The primary outcomes include the following:

#### Knowledge

This measure has been created for the purposes of this study. Adolescents will respond to 27 questions that assess their factual knowledge about clinical research (e.g., “What is a clinical trial?”). Questions are in multiple choice format (some questions have multiple correct answers), and the total score could range from 0 to 53 correct. Higher scores indicate more knowledge about clinical research.

#### Attitudes about clinical trials

This measure was originally adapted from Madsen and colleagues [28] and used in Parker et al. [26]. The items are slightly adapted. Adolescents will be asked to respond to 6 questions that assess their positive attitudes about clinical trials (e.g., How do you feel about teens participating in clinical trials?; 1 = Not good at all; 2 = Not very good; 3 = Not sure; 4 = Good; 5 = Very good; *α* = 0.83). Responses will be averaged and the minimum scale score is 1 and the maximum scale score is 5. Higher scores indicate more positive attitudes toward clinical trials.

#### Beliefs about clinical trials

Adolescents will be asked to respond to 5 questions about their beliefs about pediatric clinical research [e.g., I believe that clinical trials can help teens; 1 = Strongly disagree; 2 = Disagree; 3 = Unsure; 4 = Agree; 5 = Strongly agree; *α* = 0.78;[26]]. Responses will be averaged and the minimum scale score is 1 and the maximum scale score is 5. Higher scores indicate more positive beliefs about clinical trials.

#### Self-efficacy to communicate

This measure was originally adapted from Bandura [29] and used in Parker et al. [26]. The items were slightly adapted and split into two scales (self-efficacy to communicate and self-efficacy to gather information, below). Adolescents will be asked to respond to 10 questions about their self-efficacy for making decisions about clinical trial participation specific to communicating about clinical trials [(e.g., Tell a doctor or researcher if I want to stop the clinical trial; 1 = I cannot do it at all; 5 = I know I can do it; *α* = 0.89; [26]]. Responses will be averaged and the minimum scale score is 1 and the maximum scale score is 5. Higher scores indicate more self-efficacy about communication.

#### Self-efficacy to gather information

This measure was originally adapted from Bandura [29] and used in Parker et al. [26]. Adolescents will be asked to respond to 9 questions about their self-efficacy for making decisions about clinical trial participation specific to gathering information about clinical trials [e.g., Ask a doctor or researcher questions for more information about clinical trials; 1 = I cannot do it at all; 5 = I know I can do it; *α* = 0.89;[26]]. Responses will be averaged and the minimum scale score is 1 and the maximum scale score is 5. Higher scores indicate more self-efficacy about gathering information.

#### Confidence for participating in clinical trials

Adolescents will also be asked to respond to additional items to measure youths’ confidence for participating in clinical trials [e.g., “I know what rights I have in a clinical trial.”; 1 = Strongly disagree; 2 = Disagree; 3 = Unsure; 4 = Agree; 5 = Strongly agree; *α* = 0.77;[26]]. Responses will be averaged and the minimum scale score is 1 and the maximum scale score is 5. Higher scores indicate more confidence in participating in clinical trials.

#### Procedural fears

Adolescents will be asked to respond to 4 questions related to their perceptions of fear or anxiety about different types of medical procedures, including getting a needle in the arm, injection in the leg, getting a scan, and taking new medicine, on a 5-point Likert scale (1 = Not at all afraid or anxious, 2 = Somewhat afraid or anxious, 3 = Moderately afraid or anxious, 4 = Very afraid or anxious, 5 = Extremely afraid or anxious). Responses will be averaged and the minimum scale score is 1 and the maximum scale score is 5. Higher scores indicate more fear about procedures. The original measure has strong reliability and validity [30].

#### Likelihood of participation in clinical trials

Adolescents will be asked to respond to one question about the likelihood of participating in a clinical trial (i.e., “If you were asked to be in a clinical trial, how likely would you be to participate?”) using a 5-point Likert scale (1 = Not likely at all; 2 = Not very likely; 3 = Not sure; 4 = Likely; 5 = Very likely). Responses will be averaged and the minimum scale score is 1 and the maximum scale score is 5. Higher scores indicate greater likelihood of participation in a clinical trial.

#### Likelihood of fear preventing participation in clinical trials

Adolescents will be asked to respond to one question about the likelihood of their fear preventing them from participating in a clinical trial (i.e., “How likely is it that your fearful or anxious feelings could stop you from participating in a clinical trial in the future?”) using a 5-point Likert scale (1 = Not likely; 2 = Somewhat likely; 3 = Moderately likely; 4 = Very likely; 5 = Extremely likely). Responses will be averaged and the minimum scale score is 1 and the maximum scale score is 5. Higher scores indicate greater likelihood of fear preventing participation in a clinical trial.

#### Familiarity with clinical trials

Adolescents will be asked to respond to one question about their familiarity with clinical trials [“How much do you know about pediatric clinical trials (research studies with children under 18)?”; 1 = I don’t know anything; 2 = I know a little about them; 3 = I know some things about them; 4 = I know a lot about them; 5 = I know all there is to know about them]. Responses will be averaged and the minimum scale score is 1 and the maximum scale score is 5. Higher scores indicate greater familiarity with pediatric clinical trials.

#### Willingness to participate in a clinical trial

Adolescents will review five research protocols related to a fictitious disease (“meditis”) and respond to a question about their willingness to participate in each research study (e.g., If you had meditis, would you agree to enroll in this study?; 1 = Definitely not to 7 = Definitely yes). Responses will be averaged across the five protocols and the minimum scale score is 1 and the maximum scale score is 7. Higher scores indicate greater willingness to participate in the research studies. This measure was adapted from Brody and colleagues [31].

#### Quality of parent-adolescent communication

Adolescents will be asked to respond to 8 questions related to their perceptions of their relationship quality and communication with their parents (e.g., “My parent gives me good advice.”; “When talking to my parent, he/she tries to understand my point of view.”; 1 = Strongly disagree; 2 = Disagree; 3 = Agree; 4 = Strongly agree). Responses will be averaged and the minimum scale score is 1 and the maximum scale score is 4. Higher scores indicate more positive perceptions of relationship quality. This measure has strong reliability [32].

### Parents

#### Attitudes about clinical trials

Parents will be asked to respond to 6 questions that assess their positive attitudes about clinical trials (e.g., How do you feel about teens participating in clinical trials?; 1 = Not good at all; 2 = Not very good; 3 = Not sure; 4 = Good; 5 = Very good). Responses will be averaged and the minimum scale score is 1 and the maximum scale score is 5. Higher scores indicate more positive attitudes toward clinical trials. This measure was adapted from Madsen and colleagues [28] and used in Parker et al. [26].

#### Beliefs about clinical trials

Parents will be asked to respond to 5 questions about their beliefs about pediatric clinical research (e.g., I believe that clinical trials can help teens; 1 = Strongly disagree; 2 = Disagree; 3 = Unsure; 4 = Agree; 5 = Strongly agree). Responses will be averaged and the minimum scale score is 1 and the maximum scale score is 5. Higher scores indicate more positive beliefs about clinical trials. This measure was adapted from Parker et al. [26].

#### Likelihood of participation in clinical trials

Parents will be asked to respond to one question about the likelihood of allowing their child to participate in a clinical trial (i.e., “If your child were asked to be in a clinical trial, how likely would you be to let them participate?”) using a 5-point Likert scale (1 = Not likely at all; 2 = Not very likely; 3 = Not sure; 4 = Likely; 5 = Very Likely). Responses will be averaged and the minimum scale score is 1 and the maximum scale score is 5. Higher scores indicate greater likelihood of allowing child to participate in a clinical trial.

#### Likelihood of fear preventing participation in clinical trials

Parents will be asked to respond to one question about the likelihood of their fear preventing them from allowing their child to participate in a clinical trial (i.e., “How likely is it that your fearful or anxious feelings could stop you from allowing your child to participate in a clinical trial in the future?”) using a 5-point Likert scale (1 = Not likely; 2 = Somewhat likely; 3 = Moderately likely; 4 = Very likely; 5 = Extremely likely). Responses will be averaged and the minimum scale score is 1 and the maximum scale score is 5. Higher scores indicate greater likelihood of fear preventing their child’s participation in a clinical trial.

#### Familiarity with clinical trials

Parents will be asked to respond to one question about their familiarity with clinical trials [“How much do you know about pediatric clinical trials (research studies with children under 18)?”; 1 = I don’t know anything; 2 = I know a little about them; 3 = I know some things about them; 4 = I know a lot about them; 5 = I know all there is to know about them.]. Responses will be averaged and the minimum scale score is 1 and the maximum scale score is 5. Higher scores indicate greater familiarity with pediatric clinical trials.

#### Willingness to participate in a clinical trial

Parents will review 5 research protocols related to a fictitious disease (“meditis”) and respond to questions about their willingness to let their child participate in each research study (i.e., “If your child had meditis, would you agree to enroll them in this study?”; 1 = Definitely not to 7 = Definitely yes). Responses will be averaged across the five protocols and the minimum scale score is 1 and the maximum scale score is 7. Higher scores indicate greater willingness to allow their child to participate in the research studies. This measure was adapted from Brody and colleagues [31].

#### Quality of parent-adolescent communication

This measure was adapted from Barnes & Olson [33] and used in Scull et al. [34]. Parents will be asked to respond to 16 questions regarding their perceptions of the quality of communication with their adolescent (e.g., “If my child were in trouble, she/he could tell me”; 1 = Strongly disagree, 2 = Disagree; 3 = Agree; 4 = Strongly agree; *α* = 0.85). Responses will be averaged and the minimum scale score is 1 and the maximum scale score is 4. Higher scores indicate more positive perceptions of relationship quality.

### Participant timeline {13}

**Fig. 1:**
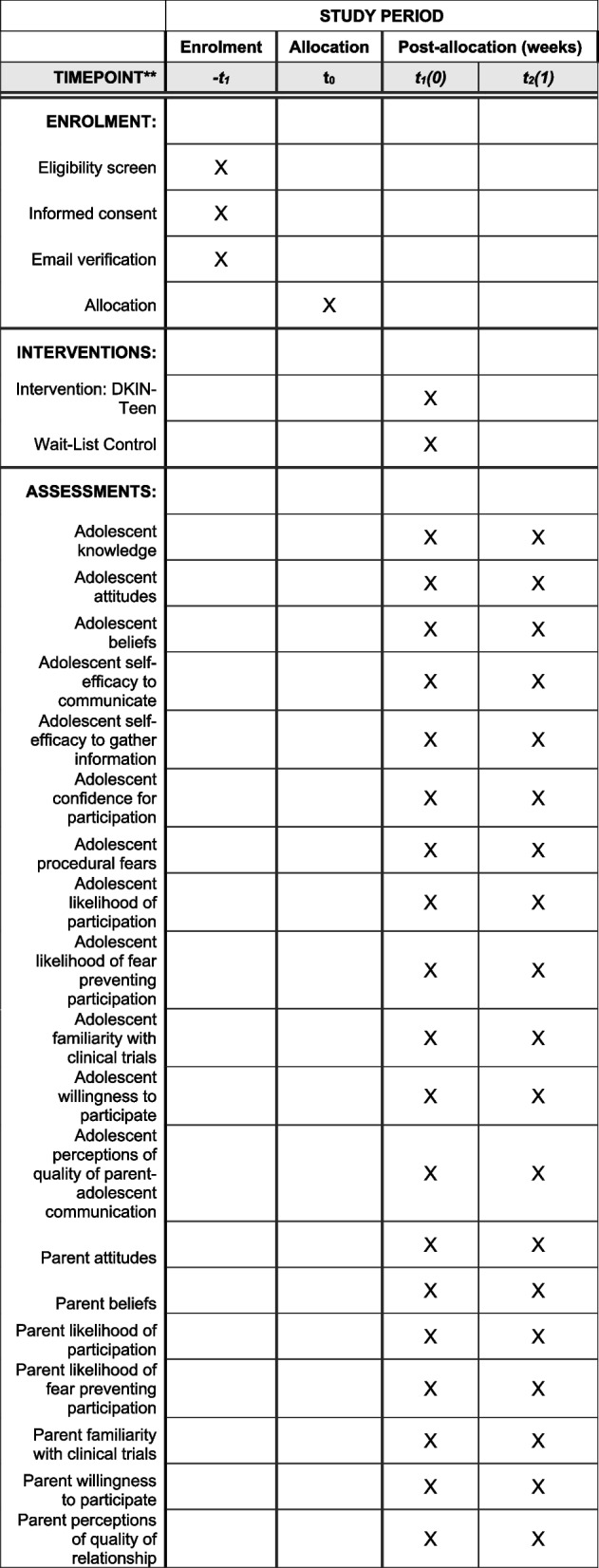
Participant timeline

See Fig. 1 for the SPIRIT figure with participant timeline.

### Sample size {14}

Based upon a 2(condition) X 2(health status) X 2(time) repeated measures design, a desired power of 0.80, a 0.30 effect size for pediatric outcomes, and an estimate of 0.7 correlation between measurements [max correlation from previous study [26]], a power analysis (*α* = 0.05) revealed approximately 144 parent-adolescent pairs are needed to participate. With an estimated attrition rate of 25% from pre-test to post-test, the aim will be to recruit 180 pairs to enroll in the study (*n* = 90 per condition).

### Recruitment {15}

Parent-adolescent pairs will be recruited from across the USA. A study recruitment website will contain information about *DigiKnowIt News: Teen* and the study that interested parent-adolescent pairs can review. The website will have the study team contact information and a fillable form to indicate interest in receiving more information on how to participate and to determine if they are eligible to participate. If eligible, parents will be notified immediately and will be provided with a link to complete the online consent, permission, and assent forms.

Parent-adolescent pairs will also be recruited via flyers shared by email and social media as well as by word-of-mouth and help from pediatric health organizations, health advocacy groups, and other parent groups and organizations that work directly with families of different races and ethnicities. School districts with diverse student populations will be identified and sent a recruitment e-flyer about the study via a digital flyer service within school districts.

### Assignment of interventions: allocation

#### Sequence generation {16a}

After consent, permission, and assent are given, participants will be randomly allocated to either the intervention (*DKIN: Teen*) or wait-list control group with stratification by gender (boy; girl), race/ethnicity (white and not Hispanic/Latino; nonwhite and/or Hispanic/Latino), and health status (healthy; chronic illness). The allocation sequence will be generated using a random number generator.

### Concealment mechanism {16b}

Recruitment will be completed in advance and participants are then randomized and enrolled based on (1) time study forms (e.g., consent) were completed, (2) confirmation of email addresses, and (3) their reported demographics collected in the eligibility screener questions. The allocation sequence will be stored in a secure folder on a secure iRT server until interventions are assigned and will only be accessible to the leading investigator (TMS) who created the sequence. The other leading investigator (AEP), who assigns participants to interventions, will only see the assignments for participants ready to enroll, and will not be able to see the future allocation sequence.

### Implementation {16c}

One of the leading investigators (TMS) will generate the allocation sequences using a random number generator in Excel. After eligibility is determined, the other leading investigator (AEP) will assign participants to condition (either intervention or wait-list control) according to those sequences and subsequently enroll participants in the study.

### Assignment of interventions: blinding

#### Who will be blinded {17a}

Participants will be blinded to assignment. All participants will receive emails to complete the pre- and post-test questionnaires. Participants in the intervention group will receive an additional email after completing the pre-test questionnaire informing them that they can review *DKIN: Teen* for 1 week. Participants in the wait-list control group will be informed that they will receive the post-test questionnaire in a week*.* Participants may be able to infer their assignment based on study tasks.

The research team will not be blinded to assignment; however, the outcome assessor (i.e., online data collection system) will be blind because the questionnaires will be web-based and not administered in person by the research team, which eliminates potential bias.

### Procedure for unblinding if needed {17b}

There will be no unblinding procedures due to the nature of the intervention. At the end of the study, all participants will have received access to *DKIN: Teen*.

### Data collection and management

#### Plans for assessment and collection of outcomes {18a}

Outcome data will be collected via pre- and post-test questionnaires that will be completed by participants using an online data collection system. Prior to the start of the study, the research team will test the online data collection system.

### Plans to promote participant retention and complete follow-up {18b}

A number of strategies will be used to promote participant retention. First, iRT’s study management system will send automatic reminders that will prompt participants to complete their questionnaires and review *DKIN: Teen* (intervention group only) in a timely manner. Second, research staff will monitor retention by examining participants’ completion of the questionnaires and interaction with the website. If participants are not responding to the automatic reminders, research staff will email or call participants directly. Third, a higher value incentive at post-test ($40 for completing the pre-test questionnaire and $60 for completing the post-test questionnaire) may increase motivation to stay in the study. Related, the wait-list group will receive access to *DKIN: Teen* at the end of the study after completing the post-test questionnaire. Finally, the short duration of the study (approximately 3 weeks) should also help increase retention.

### Data management {19}

The research team will use an iRT-developed web-based data collection system for the collection of pre-test, post-test, and consumer satisfaction questionnaire data, and implementation data via the internet. Participants will be given unique identifiers that will be assigned when they complete the questionnaires. Participants will not input their names or any identifying information when accessing the questionnaires. The web-based data collection system allows for de-identified data export, and the dataset will be exported to use for data analysis and saved on the secure iRT network, with password protection such that only authorized uses will have access to the file server. Data will be reported in an aggregate or unidentifiable form and will be coded so that the data cannot be associated directly with any individual.

### Confidentiality {27}

Electronic documentation of consent, permission, and assent will be stored on a secure iRT server separately from study data. Participants will be assigned randomly assigned unique identifiers on the questionnaires. The questionnaires will not include names or individually identifying information; thus, there will be no way to directly link individuals’ responses on the questionnaires to their names. A list linking the participant names and unique identifiers will be kept on a secure server. This list will be destroyed at the end of the study rendering the data anonymous. Participants’ responses to questionnaires and any data collected about the use of the resource will be stored separately from participant contact information.

Parent email addresses will be used during the duration of the study for contact specifically related to completing study forms (i.e., permission and assent) and accessing the questionnaires and website (*DKIN: Teen*). These email addresses will be securely stored on a password-protected server and will be kept separate from any data. In addition, there will be a separate list for contact information and incentive distribution for the research team, but it will not contain any ID numbers. A de-identified data set will be exported to use to complete data analysis, and any subsequent reports or manuscripts will not contain individually identifiable information.

### Plans for collection, laboratory evaluation, and storage of biological specimens for genetic or molecular analysis in this trial/future use {33}

Not applicable as no biological specimens will be collected during this trial.

### Statistical methods

#### Statistical methods for primary and secondary outcomes {20a}

Data will be analyzed using SAS 9.4. Preliminary analysis will include a test of equivalency between groups on demographic background characteristics using chi-squared analyses for categorical variables and *t*-tests for continuous variables. In addition, psychometric analyses will be conducted to study the reliability, validity, and distributions of key variables. Handling variables with poor reliability or validity will include modification or elimination of such variables from the analysis data sets. Variables with markedly skewed distributions will be transformed or categorized to reduce the impact of non-normality on subsequent analyses. Descriptive statistics will also be conducted, including examining the distributions, correlations, means, and standard deviations of the outcome variables.

Multiple regression analyses will be used to investigate differences in outcomes (i.e., knowledge about clinical research, attitudes and beliefs toward pediatric clinical trials, self-efficacy in making decisions related to clinical trials, likelihood and willingness to participate in clinical trials, procedural fears, communication quality) using condition (intervention/wait-list control) as the independent variable of interest. A series of multiple regressions will be used to examine if using *DKIN: Teen* impacts the post-test knowledge, attitudes, efficacy, likelihood and willingness to participate, fears, and communication quality of participants. Pre-test scores for each outcome will be included as predictor variables; thus, outcome variable means will be reported as adjusted post-test scores. Demographic variables found to be non-equivalent between groups will be included as covariates in these models. The effect sizes will be calculated by dividing the appropriate contrast parameter by the sample standard deviation of the outcome. If unexpected results are obtained, then the process data recorded by the LMS (e.g., time spent on parts of the website) and measures of satisfaction will be examined to investigate whether differing dosage or satisfaction levels can explain unexpected patterns of findings.

### Interim analyses {21b}

As this is a short-term study, there are no planned interim analyses.

### Methods for additional analyses (e.g., subgroup analyses) {20b}

Additional analyses will examine subpopulations and test age, gender, and health status (chronic illness/no illness) as potential moderators of the effectiveness of the intervention.

### Methods in analysis to handle protocol non-adherence and any statistical methods to handle missing data {20c}

Analyses will be intention-to-treat, so that participants who do not adhere to the protocol are still included in the analysis in their assigned condition. Missing data will be handled in each outcome analysis with an appropriate imputation method, and estimates and standard errors will be adjusted for imputations, if warranted.

### Plans to give access to the full protocol, participant-level data, and statistical code {31c}

There are no plans for granting public access to the full protocol, participant-level dataset, and statistical code. To ensure data confidentiality for all participants, we will make the data and associated documentation available to users only under a data-sharing agreement that provides for (1) a commitment to using the data only for IRB-approved research purposes and not to identify any individual participant; (2) a commitment to securing the data using appropriate computer technology; and (3) a commitment to destroying or returning the data after analyses are completed.

### Oversight and monitoring

#### Composition of the coordinating center and trial steering committee {5d}

The trial will be directed by the leading investigators (TMS and AEP). There is no coordinating center or trial steering committee as part of this trial. All research activities and reporting requirements will be overseen by the leading investigators. The leading investigators will supervise the work of the Research Team. At minimum, monthly project meetings with the Research Team will be conducted to provide continuous updates on staff activities, problem solve, and provide a forum for project coordination. The Research Team will be responsible for the execution of the research methodology and statistical analyses. Members will include the leading investigators, Research Specialist, and a Statistician. The Research Specialist will contribute to recruitment efforts and coordinate data collection. The Statistician will lead the statistical analyses as part of this randomized controlled trial to evaluate the effectiveness of *DKIN: Teen*. The leading investigators will assist with the analyses, and review and contribute to the findings. The leading investigators will have weekly meetings to discuss the status of the project, separate from the team meetings.

### Composition of the data monitoring committee, its role and reporting structure {21a}

A Data Monitoring Committee (DMC) is not needed because this trial protocol is a low-risk study. This trial has a Data Safety Monitoring Plan (DSMP) which includes the following: (1) Description of the project, (2) Summary of Methods for the RCT Study, (3) Overall Framework for Safety Monitoring, (4) Frequency of Monitoring, (5) Management and Reporting of Adverse Events and Unanticipated Problems, and (6) Individuals Responsible for Trial Monitoring and Advising. The DSMP includes detailed guidance on how information will be monitored. The information to be monitored from participants includes contact information, questionnaire, and implementation data from the use of the website (e.g., responses to online questions, duration of use). In addition, potential risks and protections against risk, confidentiality procedures, data collection and treatment, data storage, project monitoring, project staff procedures, server security, and reporting procedures are detailed in the DSMP. The leading investigators will meet weekly to discuss the status of the research and any possible unforeseen issues. The Research Team will report any issues that arise during the trial to the leading investigators.

### Adverse event reporting and harms {22}

This is a low-risk study. Due to the nature of the educational website and data to be collected, no study-related adverse events (AEs) are anticipated. However, any AEs that occur will be evaluated by the leading investigators and reported to the IRB promptly. Serious adverse events (SAEs) will be evaluated within 24 h, and any other unanticipated problems (UPs) or events will be evaluated within 72 h; reporting to the IRB will occur within 2 weeks. In the event of an SAE or UP, the research team will use the following reporting procedures:

1. When the research staff becomes aware of an SAE or UP, reporting requirements will be reviewed immediately and implemented in a timely manner when necessary.
2. The leading investigators will provide written documentation of the SAE or UP to the IRB Chair.
3. One of the leading investigators will send an email to the NIH Program Officer that an SAE or UP has occurred and indicate that a complete description of it and how it was handled will be detailed in a report to follow.
4. The IRB Chair will request a meeting onsite or via telephone to discuss the situation.
5. Following this discussion, the leading investigators may make recommendations to the IRB Chair about possible revisions in the study protocol, ensuring that the changes are in the best interests of the participants and the research.
6. The IRB Chair will review the revised study protocol and determine if further actions are necessary.
7. The IRB Chair will provide a report of the SAE or UP and its resolution to both the NIH Program Officer and the leading investigators.

### Frequency and plans for auditing trial conduct {23}

The monitoring process will not be independent from investigators and the sponsor as the leading investigators will be responsible for conducting and monitoring the clinical trial. All project staff members will meet monthly in order to monitor the protocol, resolve issues regarding the project, and make sure that participant safeguards are constantly being properly maintained. Questions regarding data collection will be immediately brought to the attention of the leading investigators. Research staff members will scan the incoming data on a regular basis to ensure that no identifying information has been shared in the open-ended fields and will be instructed to record any problems seen or concerns about any participants as noted from their responses. The leading investigators will review and respond to these records immediately.

### Plans for communicating important protocol amendments to relevant parties (e.g., trial participants, ethical committees) {25}

All minor protocol modifications will be approved by the IRB. Any major protocol modifications, such as broadening the scope of work, will first need approval from the NIH Program Officer and funding source, National Institute of Nursing Research (NINR), before going to the IRB for approval. Changes will be made on ClinicalTrials.gov, and participants will be informed as needed.

### Dissemination plans {31a}

The leading investigators for this study are committed to the dissemination of the trial results. The results will be presented at national or international conferences and published in a timely fashion. All final peer-reviewed manuscripts that arise from this study will be submitted to the digital archive PubMed Central. In addition, trial results will be shared with consultants, health advocacy groups, and organizations that helped with participant recruitment. The results of this trial will also be submitted to ClinicalTrials.gov.

## Discussion

Web-based platforms have been successful in targeting factors (e.g., knowledge) that might influence the decision whether to participate in a clinical trial [24, 26, 35, 36]. However, there are few web-based educational resources available that are interactive and developmentally appropriate for and relevant to adolescents that not only provides information but also skill-building (e.g., communication; shared decision-making). A previous randomized controlled trial of *DKIN* provided evidence that children who used *DKIN* experienced improvements in their knowledge about clinical trials and confidence for participating in clinical trials compared to children who did not use *DKIN* [26]. *DKIN: Teen* was created to meet the developmental needs and capabilities of adolescents. The results of the proposed evaluation study will show whether *DKIN: Teen* is able to increase knowledge, attitudes, and self-efficacy related to pediatric clinical trial participation as well as the likelihood and willingness to participate in a future clinical trial among adolescents ages 12 to 17 years old and their parents. In addition, the results will demonstrate the impact of *DKIN: Teen* on communication among parent-adolescent pairs considering clinical trial participation.

This study includes several potential strengths. The randomized control trial of *DKIN: Teen* will test not only the impact of an educational website on adolescent knowledge, attitudes, self-efficacy, and willingness to participate, but also the ability to promote communication and shared decision-making among parent-adolescent pairs considering clinical trial participation. The study presents a potential significant contribution to approaching pediatric clinical trial recruitment efforts as there is little existing literature on shared decision-making between parents and adolescents considering clinical trial enrollment. There is an increased recognition of including adolescents in the decision-making process and many adolescents want to be involved in the discussion about whether to enroll in a clinical trial [17, 19]. The study will collect both adolescent- and parent-reported data regarding *DKIN: Teen* to further understand the parent-adolescent dynamic. In addition, the content included in *DKIN: Teen* was created in collaboration with and will be evaluated by a diverse group of individuals so that the intervention may be beneficial for parents and adolescents of various age, gender, race, and ethnicity. The study will also include both healthy and chronically ill adolescents who may be eligible and interested in participating in clinical trials. To discover if the website is more helpful to youth with chronic illnesses (youth who might be more focused on their health or on clinical trials to access new treatments) compared to healthy youth, moderation analyses will be conducted to explore if there are any differences in the study outcomes based on health status. Finally, as the protocol will be conducted online, in addition to the intervention also being entirely online, the study may be able to reach a diverse group of parents and adolescents from across the USA and overcome any potential recruitment barriers related to travel and families with busy schedules. The inclusion of a diverse group of participants increases the generalizability to parents and adolescents of various age, gender, race, ethnicity, and health status (e.g., chronically ill) across the USA.

Despite the strengths of the study, there may be potential limitations. Because the questionnaires and website will be accessible online, it is possible that some participants might not complete them in a timely manner or at all. To overcome this potential barrier, reminders will be automatically sent via email (and text if opted in) about completing the questionnaires and reviewing the website if not done during the allotted timeframe. In addition, participants in the wait-list control may have the opportunity to learn about clinical trials on their own. However, there is only 1 week between completing the pre-test and post-test questionnaire so there is not a lot of time for this learning to occur. In addition, there are limited resources available that can provide adolescents and parents with information about pediatric clinical trials similar to that of *DKIN: Teen*. Moreover, adolescents with chronic illnesses or diseases will be recruited to participate. There might be some challenges with recruiting adolescents with chronic illnesses or diseases because they might be harder to reach than healthy youth (e.g., recruiting within schools) and the research team is not part of a healthcare system, thus limiting access to chronically ill patients. To overcome these potential challenges with recruitment, the research team will reach out to organizations with missions supporting various health conditions (e.g., juvenile diabetes, asthma) to share recruitment materials to increase the reach to families of adolescents with chronic illnesses, diseases, or disorders.

Pediatric clinical trials have the ability to improve quality of life and disease management among adolescents; successful clinical trial recruitment and completion contribute to scientific development and medical advancements in treating pediatric diseases [7, 37, 38]. Yet despite the demonstrated benefits of pediatric clinical research, barriers to successful recruitment remain a challenge [8]. We have identified a gap that exists among developmentally appropriate resources to educate adolescents about clinical trials and promote enrollment. In addition, there is a need for evidence-based resources that focus on the parent–child relationship and the unique circumstances of adolescents’ increased autonomy, independence, and comprehension which may lead to their greater role in the decision-making process. *DKIN: Teen* will target these new concepts related to communication and shared decision-making among parents and adolescents interested in participating in clinical trials. Should the present study demonstrate *DKIN: Teen* to be effective, parents and adolescents interested in clinical trials will have available an evidence-based educational website to support their decision-making process about whether to participate in a clinical trial.

## Trial status

The protocol is IRB number: 22–002-01-EFF, dated [May 24, 2022]. The trial is registered on ClinicalTrials.gov, [NCT05714943], with public title [Website for Adolescents About Pediatric Clinical Trials], URL: https://clinicaltrials.gov/ct2/show/NCT05714943

Secondary identifiers are iRT IRB [22–002-01-EFF] and NINR NIH R44NR019565. The date of first enrollment was June 2023. The trial is currently recruiting; recruitment is slated to be completed by August 2023. Enrollment is in the USA; the problem of study is education about pediatric clinical trials. The trial sponsor is innovation Research & Training (iRT), which may be contacted for public queries (5316 Highgate Drive, Suite 125, Durham, North Carolina, USA 27713; phone: 919–493-7700).

## Data Availability

The datasets generated and/or analyzed during the current study may be available from the corresponding author on reasonable request.

## Abbreviation

AE: Adverse event
DKIN: DigiKnowItNews
DKIN: Teen: DigiKnowItNews: Teen
DMC: Data Monitoring Committee
DSMP: Data Safety Monitoring Plan
FDA: Food and Drug Administration
IRB: Institutional Review Board
iRT: innovation Research & Training
LMS: Learning Management System
NIH: National Institute of Health
NINR: National Institute of Nursing Research
RCT: Randomized controlled trial
SAE: Serious adverse event
UP: Unanticipated problem
USA: United States of America
